# Oral Rehydration Salts, Cholera, and the Unfinished Urban Health Agenda

**DOI:** 10.3390/tropicalmed7050067

**Published:** 2022-04-29

**Authors:** Thomas J. Bollyky

**Affiliations:** Council on Foreign Relations, Washington, DC 20006, USA; tbollyky@cfr.org

**Keywords:** cholera, urban health, oral rehydration

## Abstract

Cholera has played an outsized role in the history of how cities have transformed from the victims of disease into great disease conquerors. Yet the current burden of cholera and diarrheal diseases in the fast-urbanizing areas of low-income nations shows the many ways in which the urban health agenda remains unfinished and must continue to evolve.

Cholera has played an outstanding role in the history of how cities, to quote the great urbanist Jane Jacobs, stopped being “helpless and devastated victims of disease,” and “became great disease conquerors”. Yet, the current burden of cholera and diarrheal diseases in the fast-urbanizing areas of low-income nations shows the many ways in which the urban health agenda remains unfinished and must continue to evolve.

**A Simple Solution**

Cholera and other diarrheal diseases have long been terrible killers of children in poor countries. During the 1970s, World Health Organization (WHO) officials estimated that there were about 500 million cases of diarrhea in children under the age of five each year, resulting in at least five million deaths annually [1].

In 1968, two young researchers, Richard Cash and David Nalin, were working on cholera treatments at the Pakistan Cholera Research Lab in Dhaka, Bangladesh [2]. There, Nalin and Cash successfully tested an oral solution of glucose and salt with 29 patients, building upon earlier scientific findings that sugar helps the gut to absorb new fluid. They would later conduct further tests to show that the same is true for children [3]. A few years later, an Indian physician named Dilip Mahalanabis demonstrated, in a West Bengal refugee camp, that oral rehydration was effective in responding to a cholera outbreak even outside a hospital or clinical setting, preventing the need for intravenous liquids in emergency relief circumstances [3,4,5].

Those results set in motion the development of an oral rehydration solution, which now costs a few cents per packet. This therapy may be administered at home, without the help of a nurse or physician, and has replaced the indiscriminate and unnecessary administration of antibiotics to treat diarrhea. Starting in 1979, employees at the Bangladesh Rural Advancement Committee (BRAC), a nongovernmental organization, went door-to-door in rural Bangladesh and taught 12 million mothers how to make and use the life-saving salt solution [6]. This effort took ten years and, in more recent years, the BRAC has extended the program to other poor nations [7]. WHO, UNICEF, and the U.S. Centers for Disease Control and Prevention have included oral rehydration solution in their protocols as an essential medicine to treat diarrhea [5].

As a result of these collective efforts, oral rehydration solution has saved the lives of an estimated 50 million people worldwide, the vast majority of them children in poor nations [8]. The treatment has helped reduce annual diarrheal deaths from five million to an estimated 500,000 in 2017, despite a significant percentage increase in the world’s population. Much of this decline is attributable to decades-old interventions, such as oral rehydration solution, the promotion of exclusive breastfeeding, and the more recent recommendation to use zinc for diarrhea treatment. A new oral cholera vaccine, which requires clean water and two doses given two weeks apart, has helped reduce massive outbreaks in Somalia and Yemen and the high burden of disease in the Democratic Republic of Congo (DRC) and South Sudan. In 2018, Bangladesh and India reported not a single death from the disease [9].

More than 50 years after its development, oral rehydration solution (ORS) is recognized as an important treatment for other forms of dehydration as well, including dehydration-induced kidney injury and Ebola virus disease. Much more remains to be done, however. The global adoption of ORS slowed after 1995, and more than half of the world’s children in low- and middle-income countries still do not receive cost-effective and easy-to-administer treatment [10,11]. Still, it is hard to disagree with the prestigious British medical journal *Lancet*, which in 1978, called oral rehydration treatment “potentially the most important medical advance of the twentieth century” [12].

The oral rehydration solution first pioneered by Nalin and Cash and distributed by Bangladesh’s innovative nongovernmental organizations has not only prevented millions of unnecessary deaths, but they—along with antibiotics and childhood vaccines—have also been one of a handful of cheap, lifesaving interventions that have enabled cities in developing countries to grow beyond the limits of their poverty and infrastructure [13]. This progress has occurred despite many poor countries being unable to make the same heavy investment in clean water and sanitation that accompanied earlier urbanization in wealthier nations. In doing so, these humble salts have contributed to the rise of a new phenomenon in human history: large, low-income country cities.

**Large Cities Were Once a Rich Country Phenomenon**

For most of human history, the only large cities were either wealthy industrial centers, such as Liverpool or London, or the capitals of empires, such as Rome, which could draw enough migrants from the countryside to compensate for the loss of city dwellers due to the unrelenting assault of viruses, bacteria, and parasites that accompanied crowds of people. Great epidemics like the Plague of Athens are famous for ravaging the urban centers of antiquity, but it was the everyday killers—tuberculosis, dysentery, and other intestinal and diarrheal diseases—that kept large cities deadly for millennia. As recently as 1800, only 3 percent of humanity lived in cities.

Scientific advancements, such as germ theory, and industrial and consumer demands for clean water and fire protection, all contributed to the public health revolution that followed [14,15], but it was the repeated pandemics of cholera in the 19th century that demonstrated that selective sanitation only for the wealthy was insufficient to prevent the heavy toll of water-borne disease [16]. The first municipal and national boards of health in Britain and the United States were established after repeated outbreaks of cholera [17,18,19]. Access to municipal waterworks increased exponentially, from low levels to widespread coverage over a few decades [20]. The percentage of urban American households supplied with filtered water grew from 0.3 percent in 1880 to 93 percent in 1940 [21]. In 1857, no U.S. city had a sanitary sewer system; by 1900, 80 percent of U.S. city residents were served by one [22]. Life expectancy at birth for males in New York City rose from 29 years in 1880 to 45 years in 1910 [23]. Improved access to filtered and chlorinated water alone accounted for nearly half of the decline in mortality in U.S. cities between 1900 and 1936 [24]. Similar advances were seen elsewhere in the cities of Europe and other industrializing nations [25,26].

As the relentless toll of everyday plagues and parasites lessened, more big cities emerged, but only in wealthy, industrialized nations. When the United Kingdom became one-third urbanized in 1861, the average income of its citizens was around USD 5000 (measured in 2005 dollars) [27]. The United States became a majority urban country in 1920 with a per capita income, in contemporary terms, of about USD 7500 [28].

It was not until 1960 that growth in cities started to shift to poorer nations. No low-income country with a per capita income below USD 1250 (measured again in 2005 dollars) was more than one-third urbanized in 1960; six nations with per capita incomes between USD 1500 and USD 2500 reached that threshold, almost all in Latin America [27]. Over time, the level of the wealth of nations urbanizing progressively declined, and urban population growth shifted to South Asia and sub-Saharan Africa [29]. In some cases, this urbanization has occurred ahead of the industrialization that prompted migration to cities in wealthier nations [30]. With the decline in endemic infectious diseases and child mortality, the natural growth rate has contributed a larger share to the overall increase in urbanization in low- and middle-income nations [31]. The population of city dwellers globally is projected to grow by 2.5 billion by 2050, with nearly 90 percent in lower-income nations in Africa and Asia.

**The Global Geography of Cities and the Unfinished Urban Health Agenda**

Urbanization in lower-income nations could offer billions of people better access to jobs and healthcare services and a gateway to the world economy. No country has ever become wealthy without urbanizing first. To reap those benefits, those nations will have to confront the looming health and environmental challenges of urban life [32].

Population growth is outpacing city infrastructure and the expansion of public services in the fastest-urbanizing nations, especially in sub-Saharan Africa. The availability of piped water in cities in the region fell by 10 percent between 1990 and 2015, and only four out of ten new city residents had access to improved sanitation, as defined by the World Health Organization [33]. The construction of adequate housing and paved roads is likewise not keeping up with urbanization in many poor cities in the region. Nigeria, the most populous nation in sub-Saharan Africa, is projected to have a shortfall of 20 million urban housing units by 2030 [34].

The results of urban population growth outpacing city infrastructure are slums, informal settlements where 880 million people live worldwide [35]. Poor, crowded cities with limited health systems have also been the ideal incubators for outbreaks of emerging infections, like the Ebola epidemics in West Africa in 2014 and the Democratic Republic of Congo in 2018. Both SARS-CoV-1 and SARS-CoV-2 might have originated from uncontrolled urban wet markets. Modern cities are often larger and denser than Athens and the other urban centers of antiquity; outbreaks that occur in today’s cities can spread internationally faster and with greater ease via global trade and air travel. Higher levels of air pollution are also a threat, responsible for killing an estimated 6.1 million people prematurely in 2016 [36].

The slums in lower-income nations today are considerably healthier than slums in the 19th century cities of the United States and Europe, where between 200 and 300 out of every 1000 children under the age of five died. There is limited health data on modern slums, however, and much progress is reported in averages that may mask disparities. There is some indication that the health benefits of urban life may not be equally distributed among the poor residents of cities like Cairo, Dhaka, or Nairobi [37,38].

A recent study found that cities offer greater access to piped water and sanitation, but that reported rates of diarrhea increase with greater urban density in lower-income nations [30]. Municipal water systems in dense urban areas are older, poorly maintained, and suffer from low or intermittent water pressure, which reduces the effectiveness of chlorination [39,40]. Many cities in low-income countries supply water on a rotating basis for a limited number of hours at a time [41]. Moreover, urban water systems are only effective in fighting water-borne disease when paired with street cleaning and well-functioning sewer systems, which many lower-income country cities still lack [42,43]. Waste treatment plants are rare in Africa and Asia, and treat only 15 percent of municipal wastewater in Latin America [44]. Deaths from cholera and other diarrheal illnesses in lower-income countries are generally decreasing much faster than the incidence of these diseases, suggesting that treatment is playing a large role than effective prevention [45]. Other rising health concerns also increase with urban density in lower-income nations, such as obesity, high blood pressure, and diabetes [30].

It may be time to think about the urban health agenda more as being more about economic geography than cityscapes per se [46]. This insight rings true to the lived experience of this COVID-19 pandemic. The most devastated areas in wealthy and poor nations alike have often been those with crowded living conditions, poor and socially marginalized residents, workplaces with few provisions for worker safety, and inadequate access to public services such as effective sanitation and safe water [47]. This is a particular concern in the fast-urbanizing regions where ORS is lowest in central sub-Saharan Africa, parts of western and eastern sub-Saharan Africa, the Middle East, and South America [5].

Societal and medical responses to cholera have helped shaped the history of cities, but it is cities that will define the future burden of cholera, diarrheal disease, and global health more broadly.

## Data Availability

Not applicable.
